# Low dose doxycycline decreases systemic inflammation and improves glycemic control, lipid profiles, and islet morphology and function in *db/db* mice

**DOI:** 10.1038/s41598-017-14408-7

**Published:** 2017-10-31

**Authors:** Na Wang, Xiong Tian, Yu Chen, Hui-qi Tan, Pei-jian Xie, Shao-jun Chen, Yu-cai Fu, Yi-xin Chen, Wen-can Xu, Chi-ju Wei

**Affiliations:** 1 Multidisciplinary Research Center, Shantou University, Shantou, 515063 Guangdong, China; 2 Laboratory of Cell Senescence, Shantou University Medical College, Shantou, Guangdong, 515041 China; 3 Department of Endocrinology, the First Affiliated Hospital of Shantou University Medical College, Shantou, Guangdong, 515041 China

## Abstract

The aim of this study was to determine whether low dose doxycycline as an anti-inflammatory agent could improve glucose metabolism in diabetic animals. Therefore, doxycycline was supplemented in drinking water to 6-week-old male *db/db* mice for 10 weeks. Doxycycline reduced perirenal/epididymal fat, Lee’s index, and liver cholesterol. Blood HDL-cholesterol increased, but total cholesterol and aspartate transaminase decreased. Glucose and insulin tolerances were improved, accompanying with reduced fasting blood glucose, insulin, HOMA-IR and advanced glycation end products. Islet number, β-cell percentage and mass increased, while islet size decreased. Consistently, less apoptosis but more β-cell proliferation were found in islets of treated mice. Freshly isolated islets from treated mice showed higher insulin content and enhanced glucose stimulated insulin secretion (GSIS). In addition, purified islets of Balb/c mice showed increased GSIS after cultivation *in vitro* with doxycycline, but not with chloramphenicol and levofloxacin. Inflammation markers, including lipopolysaccharides (LPS) and C-reactive protein (CRP) in serum as well as CD68-positive cells in treated islets, decreased significantly. Finally, LPS stimulated the production of inflammatory factors but inhibited GSIS of MIN6 cells; however, the effects were completely reversed by doxycycline. The results support further study of possible long-term usage of sub-antimicrobial doxycycline in diabetic patients.

## Introduction

Type 2 diabetic mellitus (T2DM) is a long term metabolic disorder that is characterized by hyperglycemia, insulin resistance, and relative insulin insufficiency. Greater release of proinflammatory mediators from obese adipose tissue has been linked to the development of insulin resistance^1,2^. Infiltration of M1 macrophages producing inflammatory cytokines including IL-6 and TNF-α that interfere with insulin signaling has been observed in obese adipose tissue^3–5^. In contrast, the anti-inflammatory M2 macrophages secreting arginase and IL-10 are the dominant-type residents in adipose tissue at lean state^1,4,6^. Recent studies have also demonstrated that a shift from M2 to M1 macrophages makes a crucial contribution to islet dysfunction in T2DM^7–9^. Further, cytokines from M1 macrophages stimulate the secretion of chemokines like IL-1β from β-cells and thus forms a vicious cycle that accelerates islet inflammation^10^.

Direct targeting inflammation may therefore have beneficial effects on glycemic control, β-cell function, and insulin resistance. Neutralization of TNF-α improves glucose homeostasis in obese rodents, but early trials using TNF-α inhibitors have yielded modest glycemic improvements in T2DM patients and obese individuals^11–13^. IL-1 receptor antagonists or monoclonal antibody against IL-1β have been demonstrated to decrease hyperglycemia, inflammatory cytokines and macrophage infiltration in rodents as well as in human^14–16^. Salsalate, a prodrug of salicylate that inhibits the NF-κB pathway, has shown promising effects on lowering glucose levels, glycated hemoglobin levels, and serum C-reactive protein (CRP) in patients with T2DM, although slight increases in low-density lipoprotein (LDL), cholesterol, and urinary albumin levels were also observed^17,18^.

In addition to their broad spectrum bactericidal activities, tetracycline and its analogues, including oxytetracycline, minocycline and doxycycline, have also been reported to possess pleiotropic non-antimicrobial effects in a myriad of diseases such as hypertension, atherosclerosis and neuropathy^19–21^. Further, tetracyclines have been implicated to possess hypoglycemic effects or could potentiated the action of insulin in both humans and animals in the 60 s and 70 s^22,23^. Recently, results from a 12-week clinical trial showed that doxycycline decreased global markers of inflammation, including CRP and myeloperoxidase (MPO), and enhanced muscle insulin sensitivity as evidenced by elevated levels of skeletal muscle phosphoinositide kinase-1 (PDK1), protein kinase B (PKB/Akt) and glycogen synthase kinase 3β (GSK3β) in obese people with T2DM^24^. A 3-month pilot clinical trial found that subjects of T2DM with periodontitis taking sub-antimicrobial-dose doxycycline obtained significant improvement at HbA1c levels as compared to placebo or subjects taking antimicrobial-dose doxycycline^25^. However, the exact mechanism underlying the hypoglycemic effects of tetracyclines is still not known.

In the present study, we carried out experiments to determine if low dose doxycycline in drinking water was able to provide beneficial effects in diabetic *db/db* mice. The results showed that doxycycline not only ameliorated insulin resistance, fasting blood glucose and insulin levels, and lipid profiles in the circulation and liver, but also improved islet morphology and increased glucose-stimulated insulin secretion. The results support further investigation of possible application of doxycycline to the treatment of T2DM.

## Methods

### Animals and The feeding experiment

Forty-five male C57BLKS/J-Leprdb/Leprdb (*db/db*) mice (5-weeks old) were purchased from the Cavens Experimental Animals of Changzhou (China) and raised in our specific pathogen-free and air-conditioned animal facility. 8–12 week-old Balb/c mice were purchased from the Shanghai Laboratory Animal Company (SLAC, Shanghai, China). Mice were fed *ad libitum* with a standard diet (SLAC, Shanghai, China) and free access to water and kept under a light-dark cycle of 12 h. After one-week adaptation period, *db/db* mice were weighted and randomly divided into three groups with 15 mice each: the control group was supplied with regular drinking water; the DC100 and DC200 groups were supplied with a concentration of 100 µg/ml and 200 μg/ml doxycycline (Sigma-Aldrich, Shanghai, China), respectively, in drinking water. Food intake and water consumption were recorded once every three days; at the same time, water was replaced to ensure doxycycline was effective. Mice were weighed individually and recorded weekly. During the 10-week experiment, 3 mice from the control and 3 mice from the DC200 group die. All study procedures and methods were approved and performed in accordance with the relevant guidelines and regulations by the Animal Care and Use Committee of Shantou University.

### Tissue preparation and measurement of physical parameters

The length of mice from the tip of nose to anus was measured using a ruler. Lee’s index (LI) was calculated using the following equation: LI = [body weight (g) × 1000/body length (cm)]^1/3^. Mice were sacrificed at week 10 after blood samples had been collected. Liver and peritoneal/epididymal fat were harvested and weighted. Livers were either fixed with 4% paraformaldehyde for hematoxylin and eosin (H&E) staining, or immediately stored at −80 °C for further experiments. Pancreases were either fixed with 4% paraformaldehyde for H&E staining, or used for islet purification with collagenase V digestion.

### Measurement of serum biochemical and inflammation parameters

Mice were anesthetized by intraperitoneal injection of pentobarbital (100 mg/kg body weight), and blood samples were drawn at 0 and 10 weeks from tail vein after food and water fasting for a minimum of 10 h. Serum was collected after blood samples were placed at room temperature for 30 minutes and spun at 3000 rpm for 15 minutes at 4 °C, and then stored at −80 °C before use. Serum immuonoreactive insulin (IRI), total cholesterol (TC), total glyceride (TG), LDL-cholesterol, alanine aminotransferase (ALT) and aspartate aminotransferase (AST) were determined using an automatic Unicel DxC 800 Chemistry Analyzer (Beckman Coulter, Brea, CA) in the First Affiliated Hospital of Shantou University. Fasting blood glucose was analyzed by using a Sannuo glucose monitor (Sannuo, China). The homeostasis model assessment-insulin resistance (HOMA-IR) index was calculated using the following equation: HOMA-IR = FSI (mU/L) x FSG (mM)/22.4, which has been reported previously^26^. Blood doxycycline concentration (Ziker Biological Technology, Shenzhen, China), advanced glycation end products (AGEs) (Luao Biological Technology, Shanghai, China), LPS (XinFan Biotechnology, Shanghai), CRP (XinFan Biotechnology, Shanghai) and lactic acid (Leagene Biotechnology, Beijing, China) were determined by enzyme-linked immunosorbent assay (ELISA) according to the instructions provided by the companies.

### Intraperitoneal glucose tolerance test (IPGTT) and Insulin tolerance test (ITT)

After an overnight fast, blood glucose levels in tail vein were measured using a Sannuo glucometer (Sannuo, China). For IPGTT, glucose concentrations were measured at 15, 30, 60, 90 and 120 min after intraperitoneal injection of a glucose load (2 g/kg). For ITT, 0.75 U/kg body weight of insulin (Novolin R, Novo Nordisk, Copenhagen, Denmark) was injected intraperitoneally, and blood glucose levels were determined as above at 0, 15, 30 and 60 min. The constant for the rate glucose disappearance of ITT (KITT) was calculated by the Lundbaek formula (0.693/half life × 100).

### Liver structure examination and glycogen staining

Liver structure was examined by regular H&E staining. The sections were mounted and examined under a microscope equipped with a CCD camera (Eclipse TE 2000, Nikon, Tokyo, Japan) using the 20x objective. Glycogen staining was carried out using the Periodic acid-Schiff (PAS) method according to the procedure provided in the PAS staining kit (Leagene, Beijing, China). Briefly, deparaffinized liver sections were rehydrated, incubated with periodic acid solution for 5 min and washed with distilled water for 5 min. Sections were covered with Schiff’s reagents for 10 min, followed by washing in running tap water for 10 min. Slides were treated with Lillie-Mayer hematoxylin and differentiated with 1% acid alcohol. Subsequently, sections were washed in tap water for 15 min until the sample turned blue, followed by dehydration with graded alcohols of increasing concentrations, cleared with xylene, mounted with neutral resins, and finally examined under a microscope.

### Measurement of liver lipids and glycogen content

To measure total cholesterol and triglyceride, 100 mg liver tissue was homogenized with 900 μl ethanol on ice and centrifuged at 2,500 rpm for 10 min. The supernatants were collected and 2.5 μl of which was mixed with 250 μl reaction solution, which includes buffer, reagents and various enzymes, provided in a hepatic total cholesterol detection kit or a triglyceride detection kit (Jiancheng, Nanjing, China) and incubated at 37 °C for 10 min, which were then measured at 510 nm in a plate reader. The contents of total cholesterol and triglyceride were calculated using pre-determined standard curves. To measure glycogen content, about 50 mg liver samples were boiled in 150 μl alkaline solution for 20 min, and 4.8 ml double distilled water (ddH_2_O) was added after cooling. The sample (0.1 ml) was then mixed with 0.9 ml ddH_2_O and 2 ml development solution provided in a glycogen detection kit (Jiancheng, Nanjing, China). The development solution should be supplemented with fresh concentrated sulfuric acid right before use as suggested in the instruction. After thorough mixing, the sample was boiled for 5 min and measured colorimetrically at 620 nm after cooling to room temperature. Finally, the content of glycogen in each sample was calculated by consulting standard tube value according to the instruction of the kit (Jiancheng, Nanjing, China).

### Determination of islet mass and size

Islet mass and size were evaluated using a previously reported morphometric method based on H&E staining^27^. Briefly, each paraffin block of the pancreas was sectioned consecutively at 5 µm thickness. A total of 30 slides (each separated by 100 μm) from each pancreas were chosen for H&E staining and examined under a microscope. To determine the area on photomicrographs, islets were circulated manually with tools in Image-Pro Plus 6.0. The relative islet mass was calculated as the sum of total islet areas. Islet numbers were counted on each slides, and islet size were calculated by dividing islet mass by islet number.

### Immunohistochemistry (IHC) and Immunofluorescence staining (IF) of pancreas

PCNA and CD68 were detected by immunohistochemistry (IHC), while insulin and glucagon were revealed by immunofluorescence staining with a previously described procedure^28,29^. Sections were incubated with the primary antibody, mouse anti-PCNA (AF0261, Beyotime, China), rabbit anti-CD68 (BA3638, Boster, China), goat anti-insulin (sc-7839, Santa Cruz, Santa Cruz, CA), or mouse anti-glucagon (BM1621, Boster, China) at 4 °C overnight, and with a secondary antibody, HRP-goat anti-mouse (Catalog#: 32230, Zymed, San Francisco, CA), HRP-goat anti-rabbit (A0208, Beyotime, China), donkey anti-goat cy3 (A0502, Beyotime, China), or goat anti-mouse FITC (A0568, Beyotime, China) for 1 h at room temperature. For IF, the nucleus was stained with DAPI (1 μg/ml, Dojindo, Japan) after washing with PBS. A total of 3 randomly chosen pancreatic sections from each mouse in each group (about 40 islets) were used for IHC or IF study. Finally, images were collected at equal exposure conditions and at the same magnification (20x objective lens) and analyzed by using the Image-Pro Plus 6.0 software.

### TUNEL staining

Terminal deoxynucleotidyl transferase dUTP nick end labeling (TUNEL) assay was carried out to determine the amount of DNA fragmentation and apoptotic cells in islets using a commercially available TUNEL kit (Beyotime, China). Briefly, deparaffinized pancreatic sections were rehydrated, washed with PBS, and incubated with 20 µg/ml DNase-free proteinase K at 37 °C for 20 min. After washing with PBS, samples were incubated in 50 µl of TUNEL reaction mixture at 37 °C for 1 h in a dark and humidified chamber, followed by three washes with PBS and addition of 10 µl of DAPI (1 µg/ml) after the last wash. One drop of mounting fluid was added to the sample before it was laid over with a cover slide and sealed with nail polish. A total of 6 randomly chosen pancreatic sections from each group (about 30 islets) were used for TUNEL staining.

### Glucose-stimulated insulin secretion (GSIS) Assay

Pancreatic islets were isolated by collagenase V digestion as previously described^29^. For *db/db* mice, islets were cultivated overnight in DMEM supplemented with 10% FBS (fetal bovine serum), 10 hand-picked islets of similar size were incubated over a period of 1 h in 100 µl glucose-free Krebs-Ringer bicarbonate (KRB) buffer, and then treated for 1 h in KRB buffer containing 2.5 mM/L or 25 mM/L glucose. The supernatants were obtained for insulin concentration determination using a rat/mouse insulin ELISA kit (Linco Research, St Charles, MO). For Balb/c mice, handpicked islets were further cultivated in DMEM medium with or without 1 μg/ml doxycycline (Sigma-Aldrich, Shanghai, China), or 100 μg/ml chloramphenicol (Meilun Biological Technology, Dalian, China), or 1 μg/ml levofloxacin (P.D. Pharmaceutical, Guandong, China) before being used for GSIS measurement. Each experiment was independently repeated three times.

### MIN6 cell culture and treatment

MIN6 cells were generously provided by Dr. Cai of NIH and have been examined routinely to be mycoplasma free. Cells were cultured in DMEM (high glucose) supplemented with 20% FBS, 1 mM sodium pyruvate, 50 μM 2-mercaptoethanol, 10 mM HEPES, 100 U/ml penicillin, and 100 μg/ml streptomycin. Measurement of insulin and cytokine secretion from MIN6 cells were performed according to a reported method with modifications^30^. Cells were cultivated in the presence or absence of 100 ng/ml LPS (Beyotime, China) for 48 h, followed by the treatment with or without indicated amount of doxycycline for 48 h. The supernatants were harvested and spun to get rid of cell debris, and then used for ELISA measurement of the inflammatory factors (TNF-α, IL-1β and INF-γ) according to the instruction provided by the company (Xinfan, Shanghai, China). For insulin secretion, cells were washed twice with 100 µl glucose-free KRB followed by preincubation for 1 h at 37 °C in 100 µl glucose-free KRB. Next, cells were washed twice with glucose free KRB prior to a 1 h incubation in 50 µl KRB including 2.5 mM or 25 mM glucose with or without doxycycline at 37 °C. At the end of the incubation, cell debris-free supernatants were used for insulin measurement with a Rat/Mouse Insulin ELISA Kit (Linco Research, St Charles, MO) according to the protocol provided by the company. Finally, cells were lysed and the supernatants were used for detection of iNOS levels with an ELISA kit (Xinfan, Shanghai, China). The levels of secreted insulin and inflammatory factors were normalized to total protein determined with the BCA Protein Assay Kit (Beyotime, China).

### Statistical analysis

All data are presented as mean ± standard error of the mean (SEM). The results were subjected to unpaired nonparametric t test. All statistical analyses were carried out using GraphPad Prizm 5. A p value less than 0.05 was considered statistically significant.

## Results

### D*b/db* mice lose fat after doxycycline treatment

**Figure 1:**
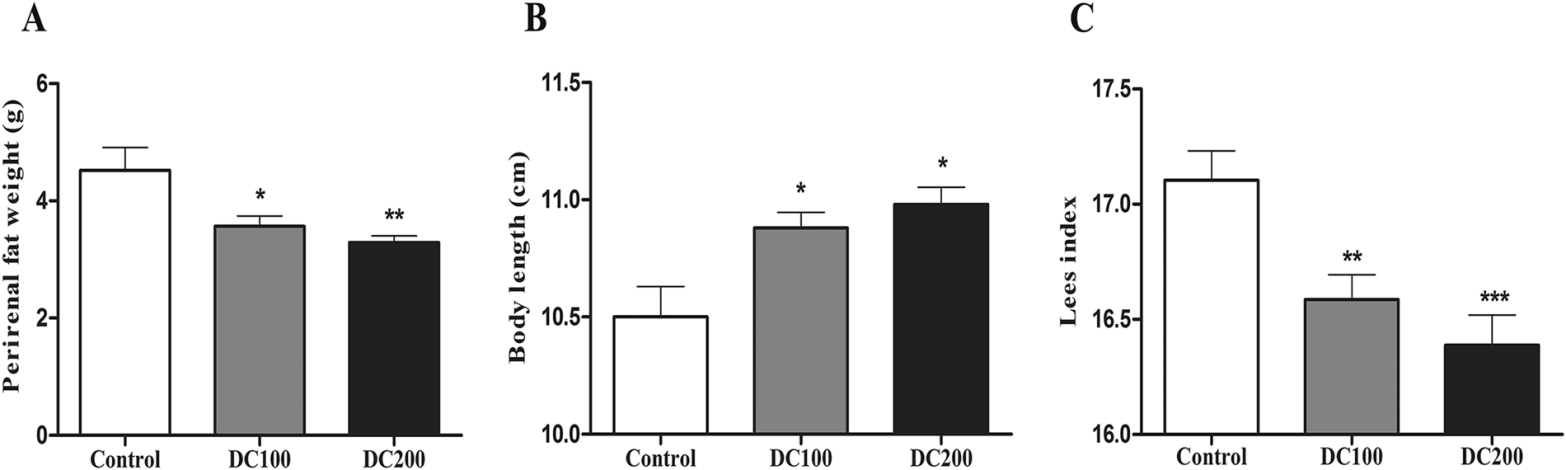
*Db/db* mice were leaner after doxycycline treatment for 10 weeks. (**A**) Perirenal/epididymal fat weight, (**B**) Body length, and (**C**) Lees index. *p < 0.05, **p < 0.01. The control group has 12 mice, DC100 and DC200 have 15 and12 mice, respectively.

Fat tissue around epididymis and kidneys reduced significantly after doxycycline treatment for 10 weeks (Fig. 1A). Moreover, body length increased slightly but significantly leading to decrease in Lee’s index (Fig. 1B/C), indicating that the mice turned leaner with doxycycline. The effects were obtained without losing body weight, food intake and water intake (Fig. S1).

### Doxycycline reduces lipid content while increases glycogen storage in liver of *db/db* mice2

**Figure 2:**
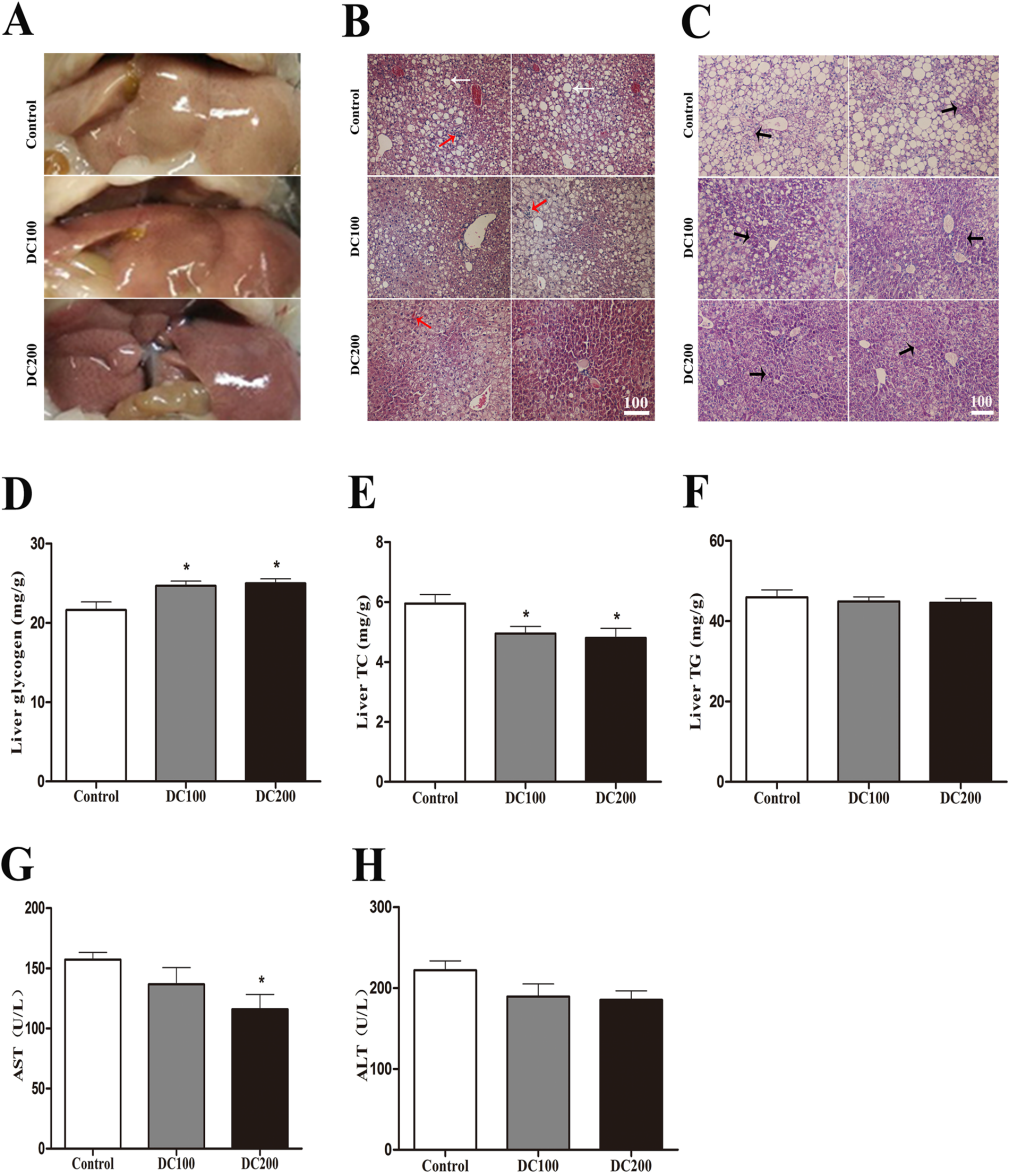
Doxycycline improved the fatty liver in *db/db* mice. (**A**) Photos of freshly harvested livers; (**B**) Photo micrographs of H&E staining; White arrow: lipid droplet; Red arrow: infiltrated inflammatory cells; CV: central vein; Scale bar = 100 μm. (**C**) Photo micrographs of PAS staining; Black arrow: glycogenosome; Scale bar = 100 μm. Liver samples were then used for quantification of liver glycogen (**D**), total cholesterol (TC) (**E**), and triglyceride (TG) (**F**); Also, serum samples were used for determination of activities of alanine aminotransferase (AST) (**G**), and aspartate aminotransferase (ALT) (**H**). *p < 0.05. n = 10.

The liver of *db/db* mice appeared yellowish, indicating of high lipid content (Fig. 2A). On the other hand, doxycycline treatment apparently reduced the degree of fatty liver, especially in DC200 group treated with 200 μg/ml of doxycycline. Of note, liver weight showed no significant differences (Fig. S2A). H&E staining confirmed the fatty liver in *db/db* mice, as evidenced by the appearance of large amount of fat deposition (Fig. 2B), which was improved after treatment with doxycycline. In addition, liver infiltration of inflammatory cells was detected in *db/db* mice, and the number of which reduced in treated mice. Similarly, doxycycline improved glycogen storage as revealed by the PAS staining (Fig. 2C). Further, the content of lipids and glycogen was quantitatively determined by a colorimetric method. The results showed that glycogen content increased by about 10%, while total cholesterol (TC) reduced by 15% in livers of doxycycline treated mice (Fig. 2D/E). However, triglyceride (TG) did not changed after the treatment (Fig. 2F). Further, the results showed that activity of aspartate aminotransferase (AST) in the circulation decreased significantly in *db/db* mice treated with 200 μg/ml of doxycycline (Fig. 2G), while that of alanine aminotransferase (ALT) reduced but not significantly (Fig. 2H), indicating that liver damage might have improved mildly.

### Doxycycline improves biochemical profiles in sera of *db/db* mice

**Figure 3:**
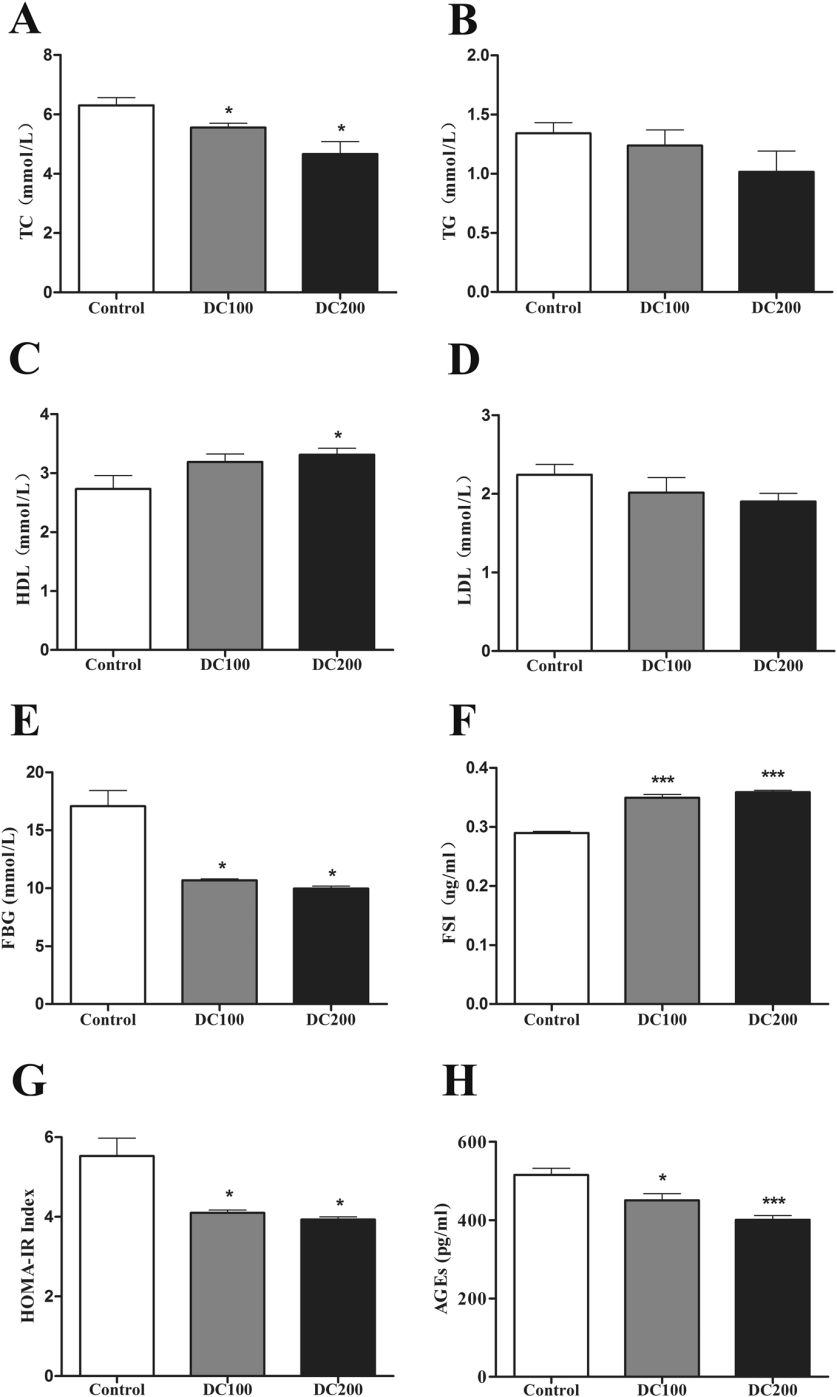
Doxycycline improves serum biochemical profiles in *db/db* mice. After food and water fasting for a minimum of 10 h, mice were anesthetized by intraperitoneal injection of pentobarbital (100 mg/kg body weight) before blood samples collection. Serum parameters were determined using an automatic Unicel DxC 800 Chemistry Analyzer (Beckman Coulter, Brea, CA) with respective reagents (Beckman Coulter) in the First Affiliated Hospital of Shantou University for (**A**) Total cholesterol (TC), (**B**) triglyceride (TG), (**C**) HDL-cholesterol, (**D**) LDL-cholesterol, (**E**) fasting blood glucose (FBG), (**F**) insulin (FSI), (**G**) The homeostasis model assessment-insulin resistance (HOMA-IR) index was calculated using the following equation: HOMA-IR = FSI (mU/L) x FSG (mm)/22.4, (**H**) Serum levels of AGEs were determined by ELISA. *p < 0.05, **p < 0.01, ***p < 0.001. n = 10.

Lipid species in the blood was improved in treated mice (Fig. 3A–D), showing that total cholesterol (TC) reduced significantly, while triglyceride (TG) and LDL-cholesterol decreased but not significantly. On the contrary, HDL-cholesterol increased significantly in *db/db* mice treated with 200 μg/ml of doxycycline.

We then measured the parameters for glucose metabolism. The results showed that fasting blood glucose dropped by about 40% in treated mice whereas insulin (FBI) increased by 16%, leading to approximately 25% reduction in HOMA-IR value (Fig. 3E–G). Eventually, advanced glycation end products (AGEs), which reflects the accumulated effects of hyperglycemia, reduced significantly in treated mice (Fig. 3H). On the other hand, the concentration of lactic acid did not changed with the administration of doxycycline (Fig. S2B), indicating that respiration rate in the mitochondria was not inhibited.

### Doxycycline improves glucose response in *db/db* mice

**Figure 4:**
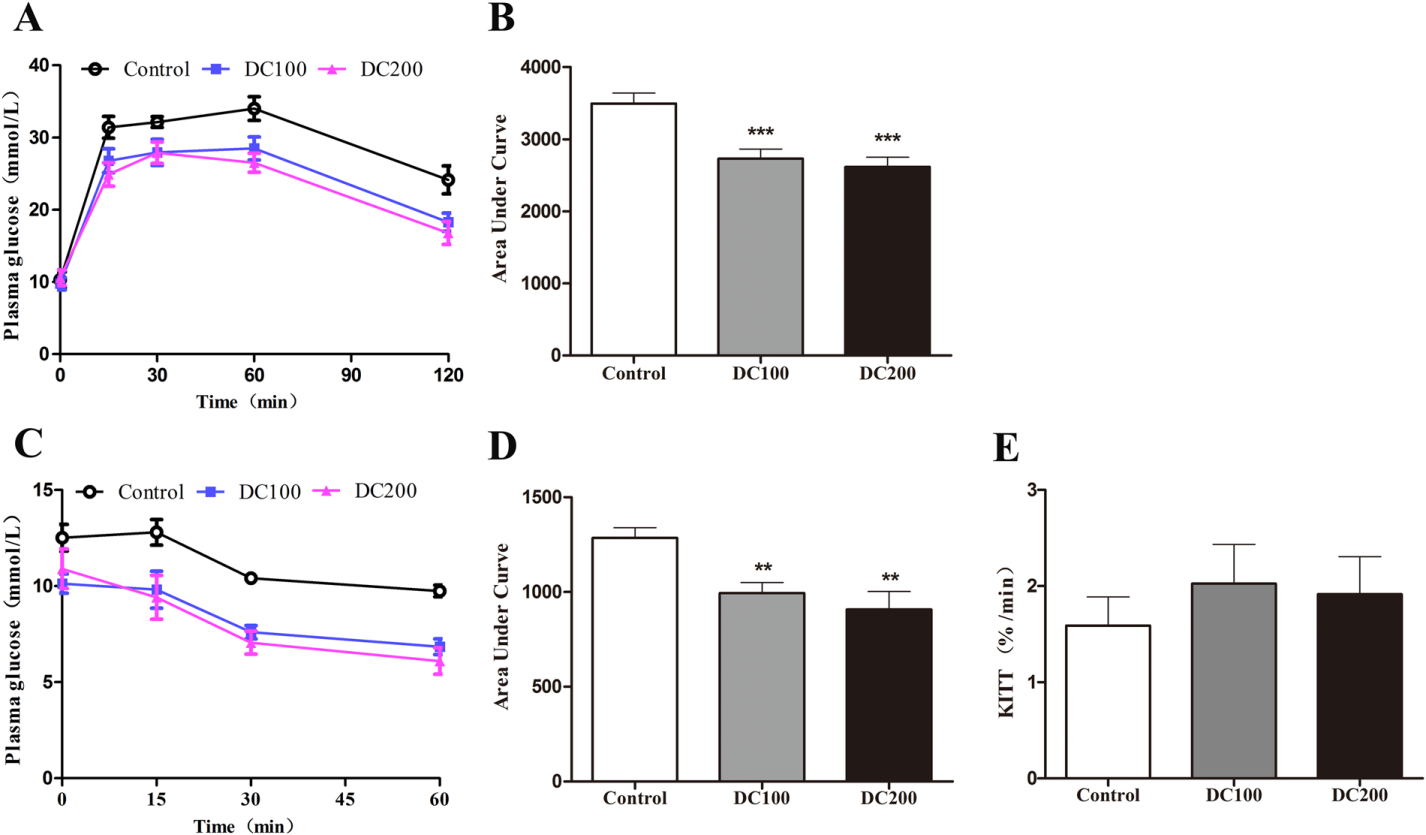
Doxycycline improved glucose and insulin tolerance in *db/db* mice. Glucose or insulin were injected via peritoneum in db/db mice after 12 h fasting, and glucose in tail blood was determined using a glucometer. (**A**) Intraperitoneal glucose tolerance test (IPGTT); (**B**) Area under the curve (AUC) for IPGTT calculated from the data in (**A**); (**C**) Insulin tolerance test (ITT); (**D**) AUC for ITT calculated from the data in (**C**); (**E**) Glucose disappearance rate of ITT (KITT) was calculated as: KITT = 0.693/t_1/2_. *p < 0.05, **p < 0.01, ***p < 0.001. n = 10–12.

Intraperitoneal glucose tolerance test (IPGTT) was then preformed, showing that glucose clearance at 30 min and thereafter was improved significantly in doxycycline treated mice (Fig. 4A). Area under the curve (AUC) of IPGTT reduced by about 20% (Fig. 4B). Similarly, insulin tolerance test (ITT) was carried out (Fig. 4C), and that AUC of ITT reduced significantly (Fig. 4D). However, when ITT was evaluated by glucose disappearance rates (KITT), the result showed no difference with the treatment of doxycycline (Fig. 4E).

### Doxycycline improves islets morphology and β-cell percentage in *db/db* mice

**Figure 5:**
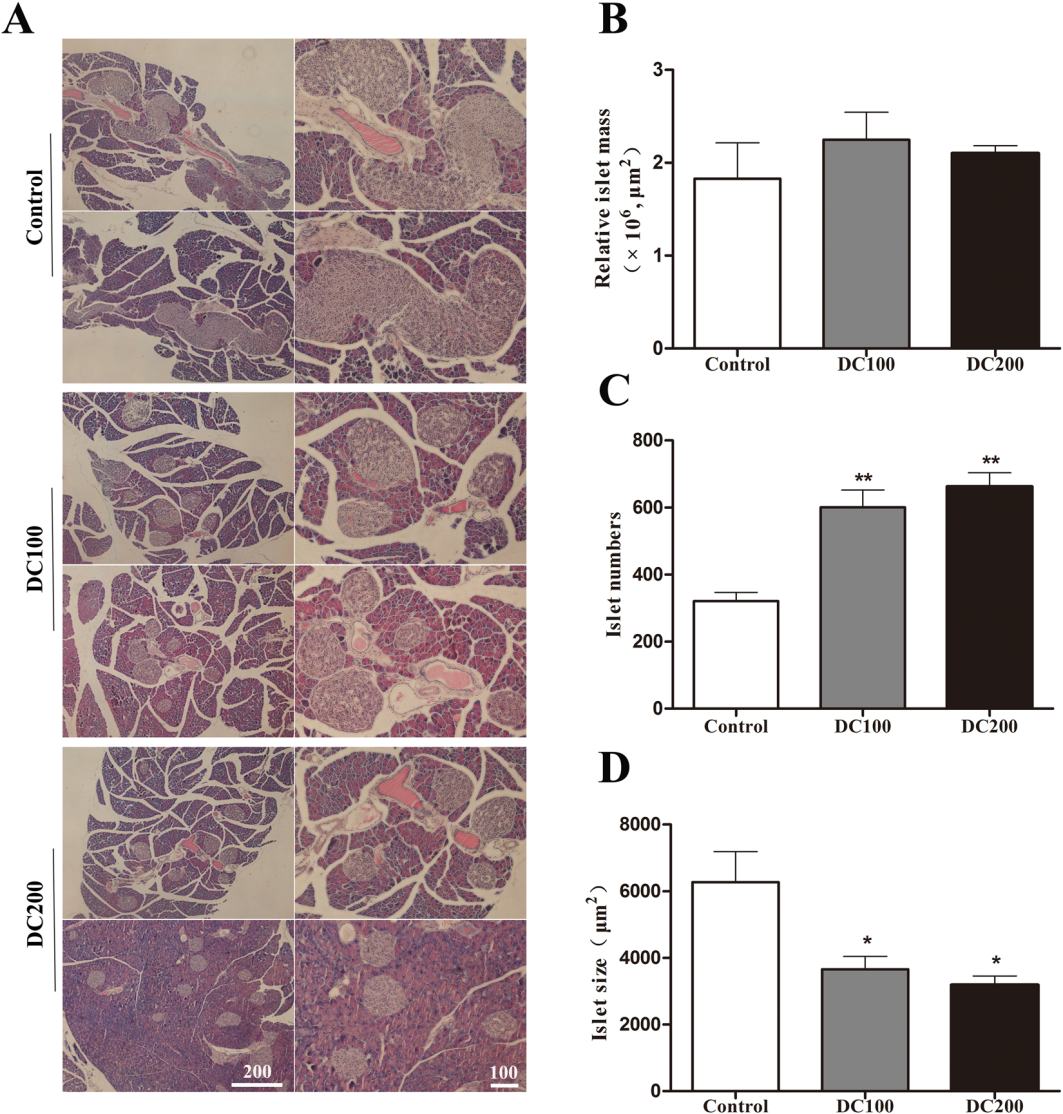
Doxycycline reduced islet size but increase islet number in *db/db* mice. (**A**) H&E staining was carried out on pancreases from each group of mice; Scale bar = 100 μm. (**B**) The relative islet mass (area) was determined using the Image-Pro Plus 6.0 software, (**C**) islet number was counted manually, and (**D**) islet size (area) was calculated by dividing islet area by islet number. Six pancreases from each group of mice were used for the quantification of islet mass, size and number. *p < 0.05, **p < 0.01.

**Figure 6:**
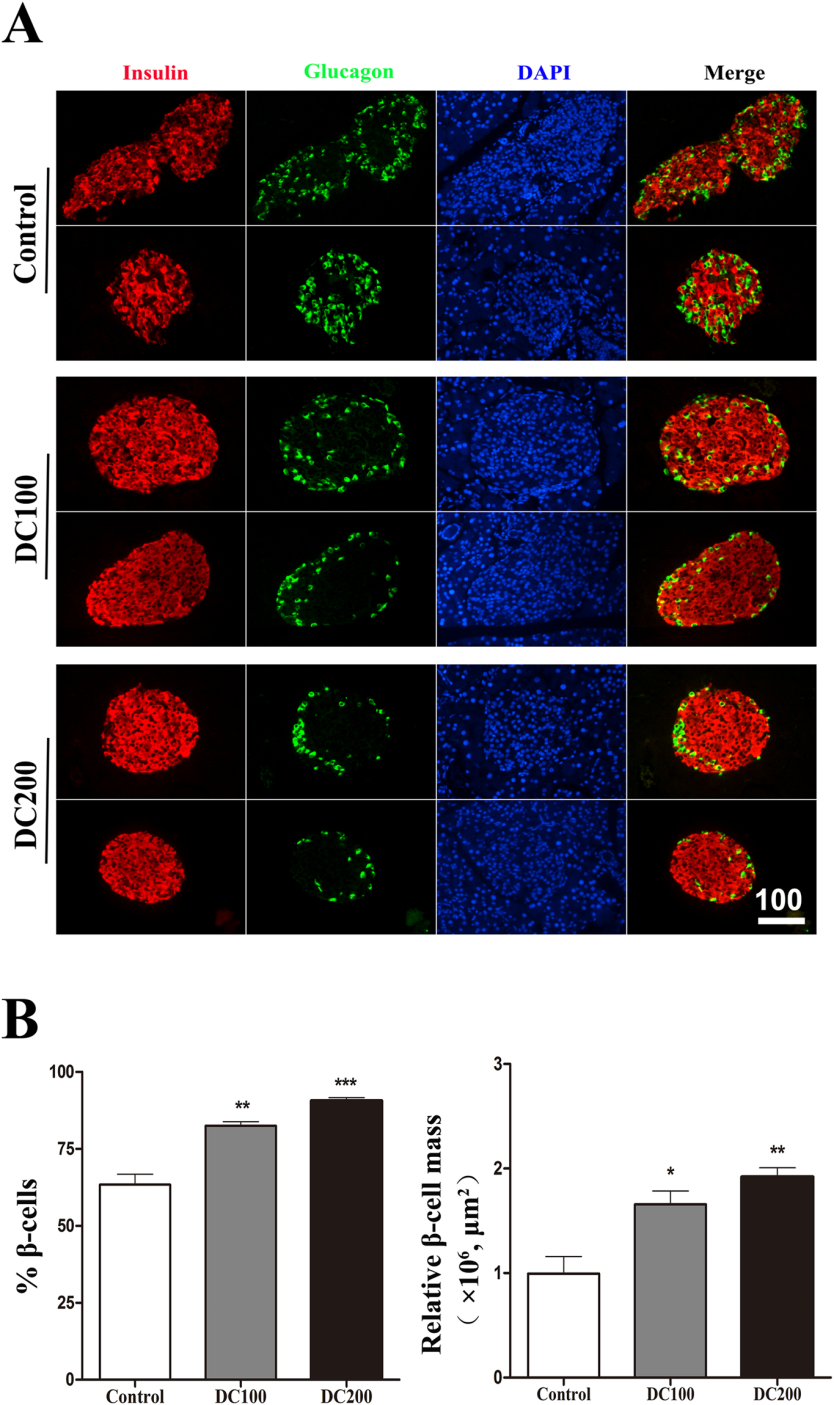
Doxycycline improved islet morphology in *db/db* mice. (**A**) Immunofluorescence staining for Insulin (red), DAPI (blue) and Glucogen (green). (**B**) Relative β-cell percentage and β-cell mass. Six pancreatic sections from each group of mice were used for the staining, and about 50 islets were measured for statistical analyses. Scale bar = 100 μm. *p < 0.05, **p < 0.01, ***p < 0.001.

H&E staining of the pancreas showed that islet size reduced dramatically in doxycycline treated mice (Fig. 5A/D). Although islet numbers increased about 1-fold, relative islet mass did not change due to the decrease of islet size (Fig. 5B/C). Immunofluorescence staining of insulin and glucagon showed that both α- and β-cells were more or less evenly distributed in the islets of *db/db* mice (Fig. 6A). Treatment with doxycycline not only increased β-cell percentage and β-cell mass (Fig. 6B), it also drove α-cells to the periphery of islets (Fig. 6A). Such a β-cell centric architecture is typically observed in the islets of Langerhans in normal mice.

### Doxycycline stimulates mitosis while inhibits apoptosis in islets of *db/db* mice

**Figure 7:**
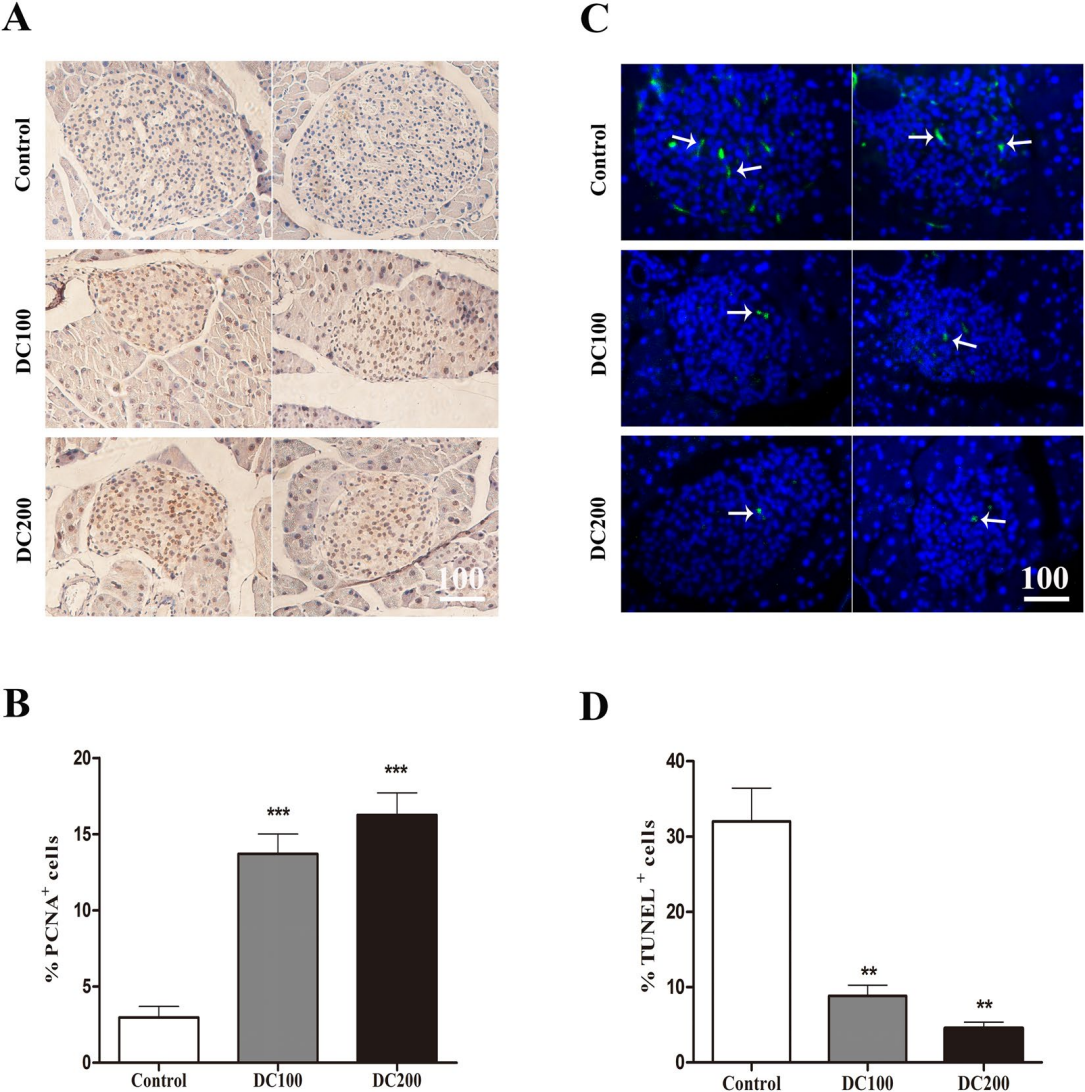
Doxycycline stimulated proliferation whereas inhibited apoptosis in islets of *db/db* mice. (**A**) IHC staining of PCNA; (**B**) Semi-quantitative analysis of PCNA positive cells; (**C**) TUNEL staining, TUNEL positive islet cells (green, arrows), DAPI (blue); (**D**) Semi-quantitative analysis of TUNEL positive cells. Six pancreatic sections from each group of mice were used for the staining, and about 40 islets were measured for statistical analyses. Scale bar = 100 μm. *p < 0.05, **p < 0.01, ***p < 0.001.

In consistent with the above observations, PCNA positive cells, indicative of the mitotic population, increased dramatically (Fig. 7A/B). On the contrary, the result from TUNEL staining showed that apoptotic cells reduced significantly in islets of *db/db* mice after doxycycline treatment (Fig. 7C/D).

### Doxycycline increases insulin content and enhances GSIS

**Figure 8:**
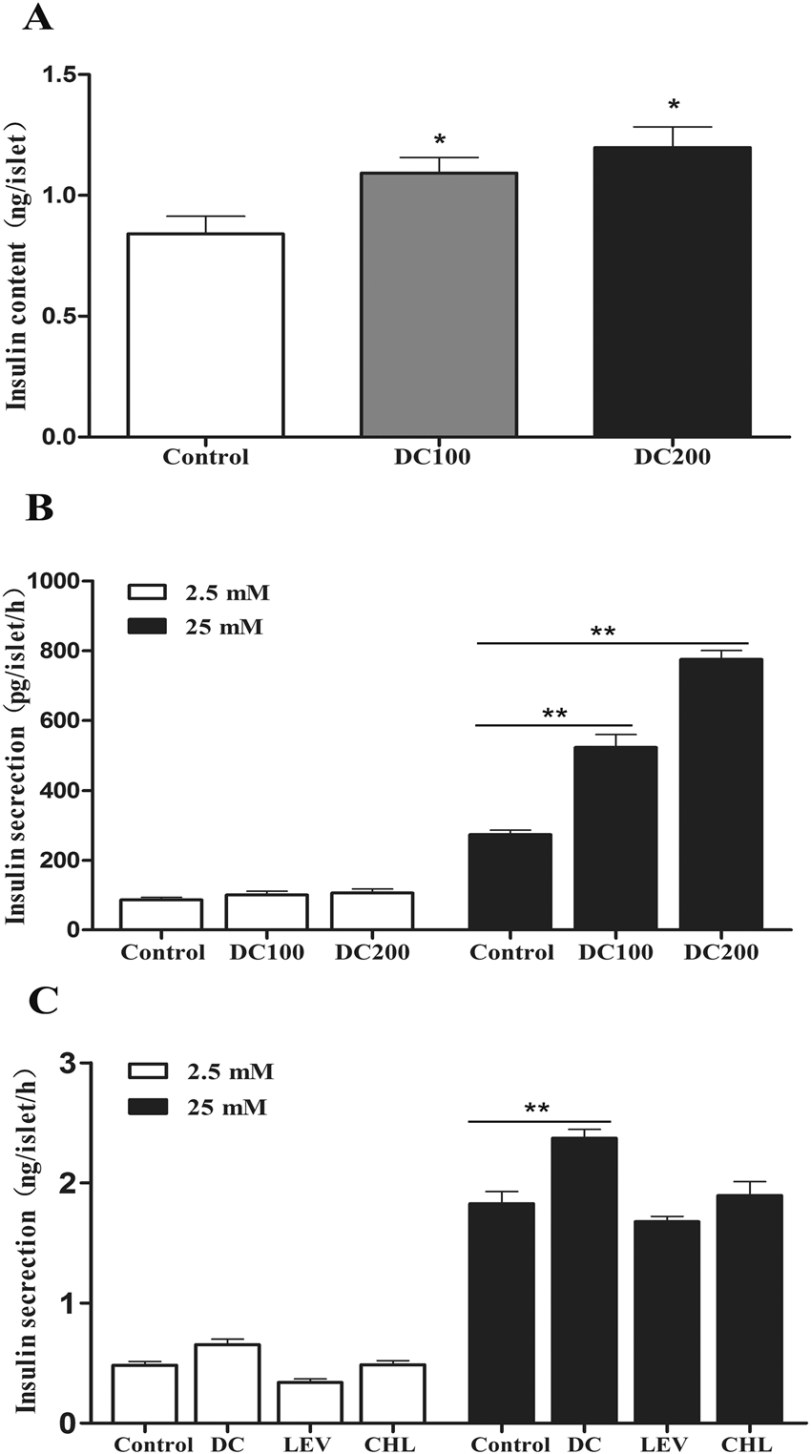
Doxycycline increased insulin content and enhanced GSIS levels. (A/B) Islets were isolated from *db/db* mice after doxycycline treatment for 10 weeks. Insulin content (**A**), and GSIS (**B**) of hand-picked islets were determined by ELISA after overnight recovery. (**C**) Islets isolated from Balb/c mice were treated with or without 1 μg/ml doxycycline (DC), 100 μg/ml chloramphenicol (CHL), or 1 μg/ml levofloxacin (LEV) for 48 h, and then used for measurement of GSIS by ELISA. Each experiment was independently repeated twice. *p < 0.05, **p < 0.01.

Islets were isolated from *db/db* mice at the end of the experiment. Treatment with doxycycline increased insulin content (Fig. 8A), and enhanced GSIS by 2–3-fold (Fig. 8B). To test if doxycycline has a direct effect, islets were isolated from Balb/c mice and incubated with doxycycline (1 μg/ml) for 48 h. The result showed that doxycycline treatment *in vitro* similarly increased GSIS by about 20% as compared to the control; while chloramphenicol (100 μg/ml) and levofloxacin (1 μg/ml) had no effect (Fig. 8C).

### Doxycycline decreases islet and systemic inflammation in *db/db* mice

**Figure 9:**
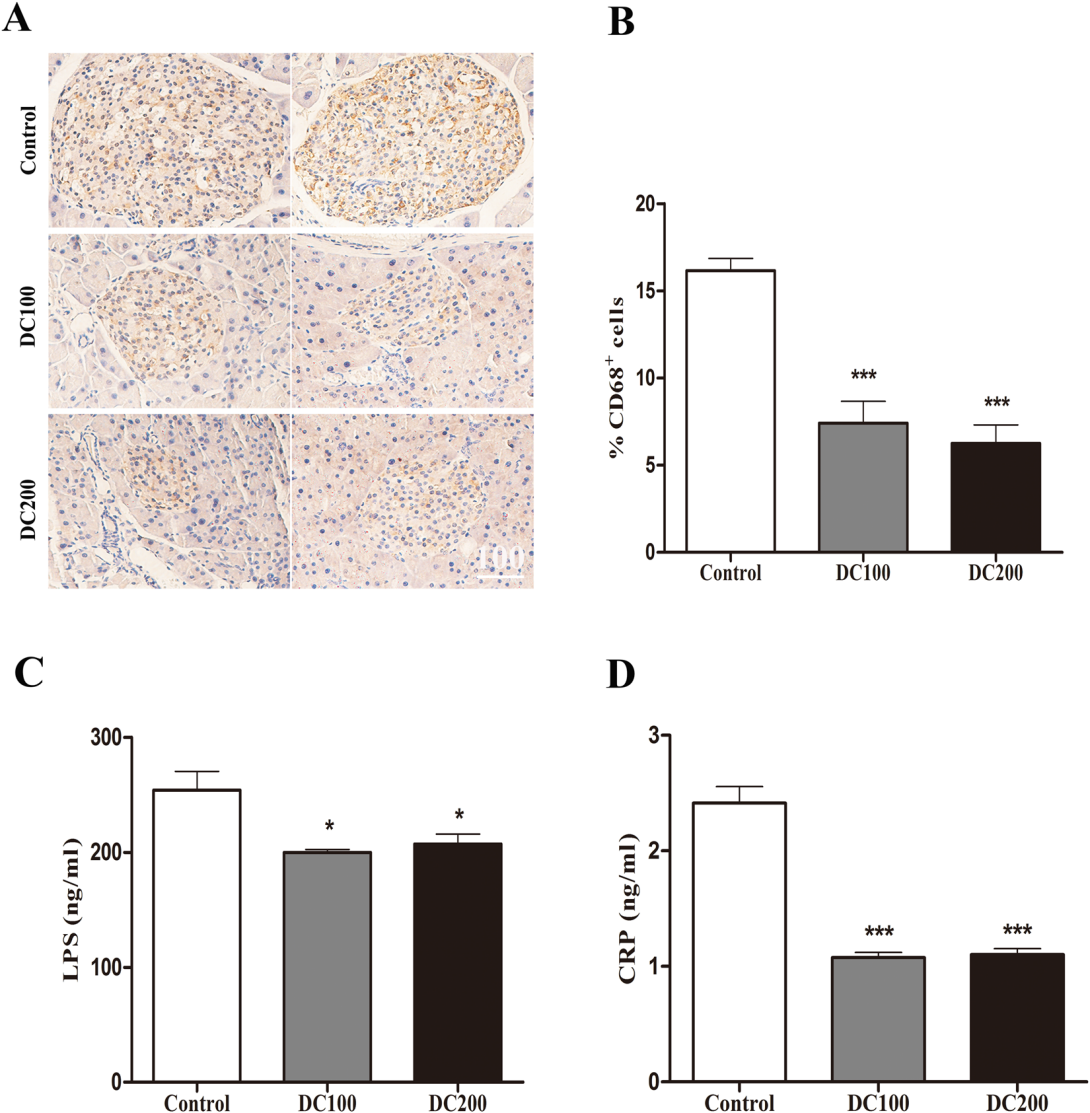
Islet and systemic inflammation in *db/db* mice was suppressed by doxycycline. IHC staining of CD68 was carried out on pancreases of *db/db* mice (**A**), and CD68 positive cells were semi-quantitatively analysed (**B**). Six pancreatic sections from each group of mice were used for the staining, and about 40 islets were measured for statistical analyses. Scale bar = 100 μm. Blood samples were treated as in Fig. 3, and LPS (**C**) and CRP (**D**) were determined by ELISA. n = 10. *p < 0.05, **p < 0.01, ***p < 0.001.

To investigate the potential mechanism underlying the hypoglycemic effect of doxycycline, islet inflammation was determined by immunostaining of CD68, which has been used as a marker of inflammatory M1 macrophages^31^. We found that CD68 positive cells reduced by more than 50% in islets of treated mice (Fig. 9A/B), indicating that intra-islet inflammation was dramatically suppressed.

Further, LPS and CRP, which reflect the extent of gut microbiota and systemic inflammation, respectively, were measured in the sera of *db/db* mice. The result showed that LPS decreased significantly but not as dramatic as the reduction of CRP (Fig. 9C/D).

### Doxycycline reverses the effects of LPS on inflammation and GSIS in MIN6 islet β-cells

**Figure 10:**
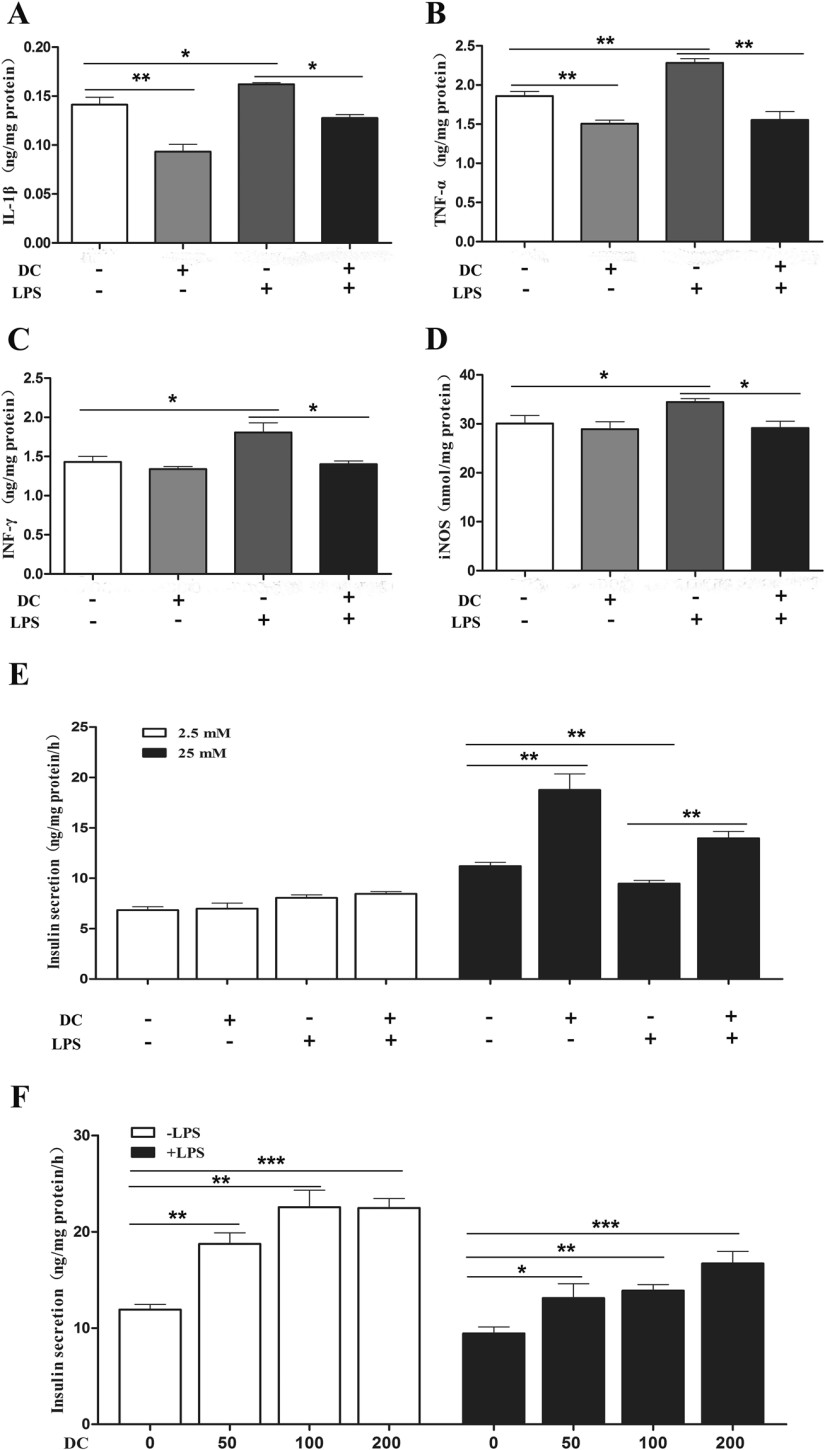
Doxycycline inhibited inflammation and enhanced GSIS of MIN6 cells. MIN6 cells were treated with or without LPS for 48 h, and then incubated with or without doxycycline for another 48 h. The supernatants were collected for measurement of inflammatory cytokines by ELISA, and cells were used for further GSIS measurement, and finally cells were lysed and the supernatants were used for iNOS measurement by ELISA and total protein measurement using BCA. (**A**-**D**) IL-1β, TNFα, INFγ and iNOS of MIN6 cells treated with 1 μg/ml of doxycycline (DC); (**E**) GSIS of MIN6 treated with 1 μg/ml of doxycycline at 2.5 and 25 mM of glucose; (**F**) GSIS of MIN6 treated with 0–200 ng/ml of doxycycline (DC) at 25 mM glucose. Each experiment was carried out in triplicate and independently repeated three times. *p < 0.05, **p < 0.01, ***p < 0.001.

We then used MIN6 islet β-cells to test the anti-inflammatory effect of doxycycline directly. The results showed that incubation with LPS significantly increased the production of iNOS and the secretion of IL-1β, TNFα and INFγ (Fig. 10A–D), while further treatment with doxycycline completely abrogated the inflammatory effect of LPS. Of note, in the absence of LPS, doxycycline also decreased the basal secretion of IL-1β and TNFα.

Finally, we tested the effect of doxycycline on GSIS of MIN6 cells. The result showed that insulin secretion increased in the presence of doxycycline while significantly reduced in the presence of LPS (Fig. 10E). Further, the inhibitory effect of LPS on GSIS of MIN6 was abrogated by doxycycline. Moreover, when titrated down to 50 ng/ml, doxycycline was still able to stimulate GSIS and reverse the inhibitory effect of LPS on MIN6 cells (Fig. 10F). Of note, the concentration of 50 ng/ml was lower than serum doxycycline in DC100 and DC200 mice, which was found in the range of sub-bactericidal concentrations of 83–144 ng/ml.

## Discussion

The results in the present study confirmed the hypoglycemic effects of doxycycline even provided in low dose in drinking water. Administration of doxycycline in *db/db* mice for 10 weeks led to improved glucose tolerance and insulin sensitivity (Fig. 4), accompanying with reduced fasting blood glucose, insulin and HOMA-IR (Fig. 3E/G). The improved diabetic conditions were collectively reflected in the reduction of AGEs levels (Fig. 3H). It is noteworthy that 100–200 μg/ml of doxycycline in drinking water only resulted in a sub-bactericidal serum concentration of 83–144 ng/ml. Typically, following a normal dosage of 200 mg doxycycline for the treatment of acute infections in adults, the average peak plasma doxycycline concentration reaches about 3 μg/ml at 3 h, and reduces to about 1 μg/ml at 24 h^32^. Subantimicrobial dose doxycycline (20 mg twice daily, serum concentration might be in the range of 200–600 ng/ml), which has been routinely applied in periodontal therapy, has been repeatedly demonstrated to be safe with no evidence of microbiologic resistance occurred after the treatment^33^.

We found food and water intake of mice was maintained during the experiment (Fig. S1), which was contrary to the results of previous studies using high dose tetracyclines^34,35^. It seems like a therapeutic dosage of antibiotics, whether in drinking water or injected via muscle, lowers animal appetite probably due to a non-specific toxic effect^36,37^. On the other hand, a wide variety of antimicrobial agents at subtherapeutic doses have been demonstrated to have growth promotion effects^38^. A recent study found that administration of subtherapeutic antibiotics, including an extreme low dose oxytetracycline, increased adiposity in young C57BL/6 mice and increased hormone levels related to metabolism^39^. However, the body weight of *db/db* mice in this study did not change significantly (Fig. S1), suggesting that absorption of energy was normal with the treatment of doxycycline. Further, blood lactic acid levels were comparable (Fig. S2), indicating that mitochondrial respiration rate was not inhibited. However, the mice were leaner after the treatment as evidenced by a lower Lee’s index (Fig. 1C). In addition, less peritoneal/epididymal fat (Fig. 1A) was found and reduced liver lipid content (Fig. 2E) was detected in treated mice. Consistently, the lipid profiles in the blood were significantly improved with decreased levels of LDL-cholesterol (not significant), total cholesterol (TC) and triglyceride (TG, not significant), but higher HDL-cholesterol (Fig. 3). The results demonstrated that a large faction of energy intake was converted to body growth rather than fat storage with the administration of doxycycline.

A growing appreciation has been established for the role of microbiota in the development of obesity, insulin resistance and diabetes^40,41^. Further, accumulating evidences have found the association of LPS-producing bacteria with obesity;^42^ however, doxycycline treatment resulted in only moderate decrease of LPS in the serum (Fig. 9C). The result raised the possibility that doxycycline might exert anti-obesity and anti-diabetes functions through its anti-inflammatory activity rather than acts as an antibiotic alone. Consistent with this hypothesis, it has been demonstrated that gut-derived LPS stimulates adipose macrophage accumulation but is not essential for the development of glucose or insulin intolerance in mice^43^. On the other hand, the systemic inflammatory marker CRP reduced dramatically after doxycycline treatment (Fig. 9D). In addition, *in vitro* cultivation of purified islets from Balb/c mice showed that insulin content and GSIS was improved in the presence of doxycycline, but not the control antibiotics chloramphenocal and Ciprofloxacin (Fig. 8C). Moreover, doxycycline was found to inhibit the production of iNOS and the secretion of IL-1β, TNFα and INF-γ (Fig. 10A-D), which are critical factors for the development of dysfunction and apoptosis of islet β-cells, but increased GSIS of MIN6 cells in the presence or absence of LPS (Fig. 10E/F). Even though the role of anti-inflammatory activity of doxycycline has been well appreciated, and that several potential molecular targets have been identified^21,44^, the exact mechanism is still not clearly understood.

Another striking observation of the present study was the small islet size but large number of islets in doxycycline treated pancreases (Fig. 5). Administration of doxycycline also reduced the apoptosis of islet β-cells (Fig. 7C/D), increased β-cell mitotic activity (Fig. 7A/B) and percentage (Fig. 6B), and improved islet morphology including the β-cell centric arrangement (Fig. 6A). In addition, treatment of doxycycline reduced islet inflammation as demonstrated by less CD68 positive macrophages (Fig. 9A/B), which probably represents the M1 pro-inflammatory population^31^. Further, after the treatment, islets from *db/db* mice showed higher insulin content and GSIS (Fig. 8A/B). Contrary to our results, islet size turns larger after the medication of metformin or pioglitozone in diabetic animals^45,46^. It has been reported that islets in *db/db* mice become abnormally larger and larger as the animals age apparently in respond to the greater demand of insulin to counter the increasingly higher glucose levels in the blood^47^. Theoretically, islet mass in adult pancreas could increase via two strategies: islet neogenesis and β-cell replication;^48,49^ however, we found the latter to be the predominant phenomenon in mice after one month old under physiological conditions^29^. Since the ratio of islet mass to body weight remains constant in adult pancreas^29^, the results of present study suggested that doxycycline seemed able to stimulate islet neogenesis as well as β-cell replication in *db/db* mice.

In conclusion, the results from the present study demonstrated that low dose doxycycline in drinking water had anti-obesity and anti-diabetes functions. Although the exact mechanism is still not clearly known, the anti-inflammatory activity of doxycycline probably is indispensable. The unique features found in doxycycline treated *db/db* mice, especially the maintenance of small islet size, warrants a further study of possible long-term usage of sub-antimicrobial doxycycline in diabetic patients.

### Data availability

The datasets generated and/or analysed during the current study are available from the corresponding author on reasonable request. All data generated or analysed during this study are included in this published article (and its supplementary information files).

## Electronic supplementary material

Figure S1

Figure S2
