# Supplementary material for: Low dose doxycycline decreases systemic inflammation and improves glycemic control, lipid profiles, and islet morphology and function in db/db mice
Source: Sci Rep. 2017 Oct 31;7:14707. doi: 10.1038/s41598-017-14408-7 (PMC5666019; doi:10.1038/s41598-017-14408-7)
Supplement: Supplementary file 1 — Figure S1 [file 41598_2017_14408_MOESM1_ESM.pdf]

Low dose doxycycline decreases systemic inflammation and improves glycemic control, lipid profiles, and islet morphology and function in *db/db* mice

Na Wang[1], Xiong Tian[1], Yu Chen[1], Hui-qi Tan[1], Pei-jian Xie[1], Shao-jun Chen[1], Yu-cai Fu[2], Yi-xin Chen[3], Wen-can Xu*[3], Chi-ju Wei*[1].

Address:

[1] Multidisciplinary Research Center, Shantou University, Shantou 515063, Guangdong, China

[2] Laboratory of Cell Senescence, Shantou University Medical College, Shantou, Guangdong 515041, China

[3] Department of Endocrinology, the First Affiliated Hospital of Shantou University Medical College, Shantou, Guangdong 515041, China

***Corresponding authors:**

Wei, Chi-ju (Wei, CJ, PhD):

Tel: +86-754-86503784, Fax: +86-754-82901175, E-mail: chijuwei@stu.edu.cn.

And : Xu, Wen-can (Xu, WC, PhD)

Tel: +86-754-88905428, Fax: +86-754-88259850, E-mail: xuwcan@163.com

Runing title: Doxycycline improves glucose and lipid metabolism in *db/db* mice

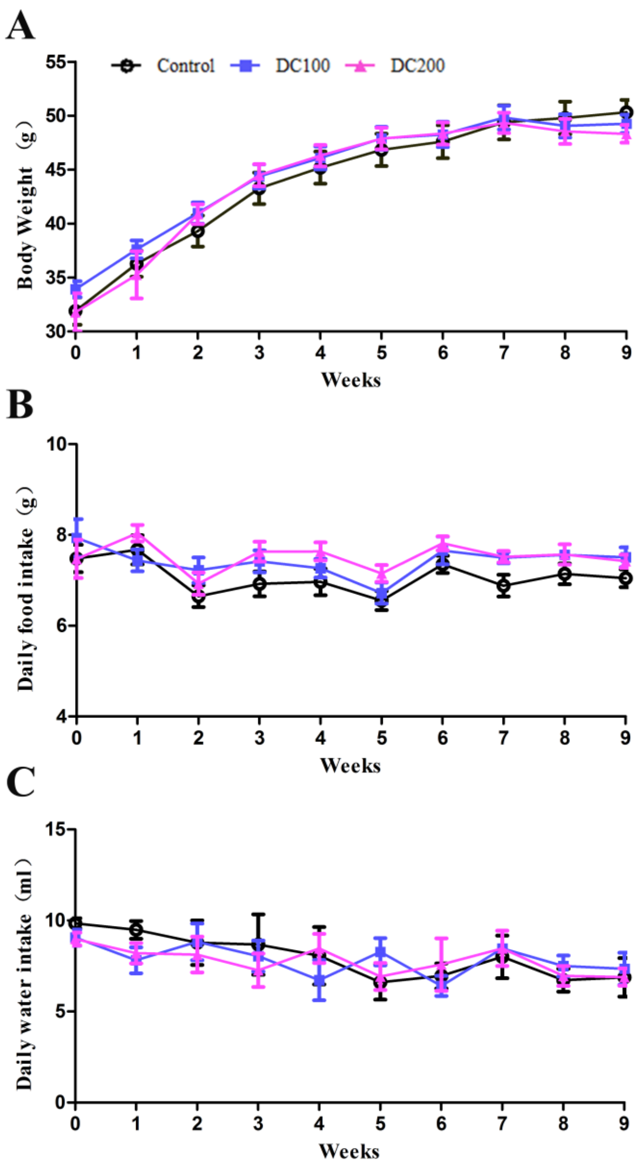

Supplementary Figure S1 Doxycycline did not affect body weight, food intake and water intake. Mice were weighed weekly, and Food intake and Water intake were measured every week, which were then divided by time (days) and number of mice in each cage. Average body weight (A), daily food intake (B), and daily water intake (C) per *db/db* mouse during week 1 to week 9. n = 12-15.
