# Supplementary material for: Low dose doxycycline decreases systemic inflammation and improves glycemic control, lipid profiles, and islet morphology and function in db/db mice
Source: Sci Rep. 2017 Oct 31;7:14707. doi: 10.1038/s41598-017-14408-7 (PMC5666019; doi:10.1038/s41598-017-14408-7)
Supplement: Supplementary file 2 — Figure S2 [file 41598_2017_14408_MOESM2_ESM.pdf]

Low dose doxycycline decreases systemic inflammation and improves glycemic control, lipid profiles, and islet morphology and function in *db/db* mice

Na Wang[1], Xiong Tian[1], Yu Chen[1], Hui-qi Tan[1], Pei-jian Xie[1], Shao-jun Chen[1], Yu-cai Fu[2], Yi-xin Chen[3], Wen-can Xu*[3], Chi-ju Wei*[1].

Address:

[1] Multidisciplinary Research Center, Shantou University, Shantou 515063, Guangdong, China

[2] Laboratory of Cell Senescence, Shantou University Medical College, Shantou, Guangdong 515041, China

[3] Department of Endocrinology, the First Affiliated Hospital of Shantou University Medical College, Shantou, Guangdong 515041, China

***Corresponding authors:**

Wei, Chi-ju (Wei, CJ, PhD):

Tel: +86-754-86503784, Fax: +86-754-82901175, E-mail: chijuwei@stu.edu.cn.

And : Xu, Wen-can (Xu, WC, PhD)

Tel: +86-754-88905428, Fax: +86-754-88259850, E-mail: xuwcan@163.com

Runing title: Doxycycline improves glucose and lipid metabolism in *db/db* mice

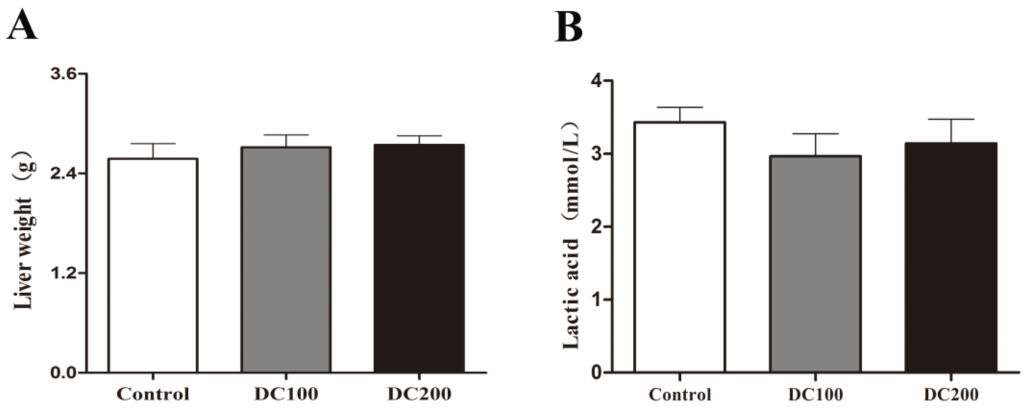

Supplementary Figure S2 Doxycycline did not affect liver weight and lactic acid levels. Mice were sacrificed after serum collection at week 10. (A) liver weight was recorded immediately after harvest. n = 12-15. (B) Serum lactic acid levels were determined by ELISA. Data are represented by mean ± sem. n = 10.
